# Comparison of Central Macular Thickness Measured by Three OCT Models and Study of Interoperator Variability

**DOI:** 10.1100/2012/842795

**Published:** 2012-08-22

**Authors:** Zaïnab Bentaleb-Machkour, Eléonore Jouffroy, Muriel Rabilloud, Jean-Daniel Grange, Laurent Kodjikian

**Affiliations:** ^1^Department of Ophthalmology, Croix-Rousse University Hospital, Claude Bernard University, 69004 Lyon, France; ^2^Department of Biostatistics, Hospices Civils de Lyon, 69000 Lyon, France

## Abstract

*Purpose*. To compare central macular thickness (CMT) measurement on healthy patient using 3 different OCT devices by two operators. *Methods*. Prospective, monocentricstudy. Right eye's central macular thickness (CMT) of 30 healthy patients has been measured three times using a time-domain (TD) OCT (Stratus OCT, Carl Zeiss Meditec, Dublin, Ca) and two spectral domain (SD) OCTs (Cirrus HD-OCT, Carl ZeissMeditec, Dublin, Ca) and 3D-OCT 1000 (Topcon, Tokyo, Japan) by two operators. Six measurements were taken randomly for each patient the same day. *Results*. No significant difference between measurements obtained by the two operators has been observed, whatever the studied OCT. *P* value was 0.164, 0.193, and 0.147 for Stratus OCT, Cirrus HD-OCT and 3D-OCT, respectively. Mean CMT significantly differed from instrument to instrument (*P* < 0.001) and was, respectively, 197 **μ**m, 254 **μ**m, and 236 **μ**m using Stratus OCT, Cirrus HD-OCT, and 3D-OCT 1000. Using Cirrus OCT and 3D-OCT 1000, CMT was, respectively, 57 **μ**m and 39 **μ**m thicker than using Stratus OCT (*P* < 0.05). *Conclusions*. Whatever the OCT device, on healthy patients CMT was not operator dependent. CMT measurements obtained by SD-OCTs are greater than those obtained by TD-OCT. These data imply that the different OCT devices cannot be used interchangeably in clinical monitoring.

## 1. Introduction

Introduced in 1991 [1], Optical Coherence Tomography (OCT) is a fundamental diagnostic tool in monitoring patients with macular disorders like diabetic retinopathy [2, 3] or neovascular age-related macular degeneration [4]. It allows assessing noninvasively morphologic changes during therapy by analyzing macular thickness.

First generation of OCT or time-domain OCT (TD OCT) [1] uses an infrared light source which is split into two separate beams. One beam is scanning a tissue being analyzed, and the other one acts as a reference beam which is reflected by a reference mirror.

Spectral domain OCT (SD OCT) [5, 6] technology uses low-coherence interferometry to detect light echoes, relying on a spectrometer and high-speed camera and based on the mathematical premise of Fourier transformation.

Recent studies showed that retinal thickness measurement differences between SD-OCT and TD-OCT devices may exist. When comparing Stratus OCT and Topcon 3D-OCT 1000 models, Leung et al. [7] found a difference of 20.8 *μ*m in macular thickness measurements; the highest ones were given by new generation of OCT. Wolf-Schnurrbusch et al. [8] compared macular thickness obtained by 6 different OCTs. Measurements obtained with Stratus OCT showed the lowest values, whereas measurements obtained with Cirrus HD-OCT and Spectralis HRA+OCT yielded the highest ones. Intermediate measurements were obtained with Copernicus, SLO and RTVue-100 OCTs.

The purpose of our study was to demonstrate differences in central macular thickness (CMT) measurements generated by different SD- and TD-OCT instruments and by two different operators. For this purpose, we compared CMT measurements generated by the Stratus OCT (Carl Zeiss Meditec, Dublin, Ca, USA), the Cirrus HD-OCT (Carl Zeiss-Meditec), and the Topcon 3D OCT 1000. Additionally, to study interoperator variability we compared measurements obtained by each operator.

## 2. Methods

### 2.1. Study Population

In this prospective study, CMT was assessed at various time from 2009 May to 2009 November in 30 right eyes of 30 healthy volunteers from the staff of our department by 2 operators (Z.M-B and E.J.), with similar practical OCT experience.

Inclusion of the thirty participants was based on a complete ophthalmologic examination including a visual acuity test, an IOP measure, and an anterior segment examination through a slit-lamp and a fundus biomicroscopy through a nondilated pupil.

Exclusion criteria were a corrected visual acuity fewer than <20/20, glaucoma antecedent, ocular hypertension, diabetes, blood hypertension, amblyopia, ocular surgery, or abnormalities during the ophthalmologic examination.

Measurements were performed on the same day, in a random order. All subjects underwent OCT imaging using each of the three OCT devices at various times from 2009 May to 2009 November. This leads to six measurements by subject.

### 2.2. Optical Coherence Tomography Imaging

Central macular thickness was determined automatically and was analyzed by OCT software. The pupil was not dilated. In all OCT maps, automated macular thickness detection was performed automatically by instrument's software analysis without manual operator adjustment.

Stratus OCT images were generated using the Fast Macular Thickness Scan consisting of six radial scans oriented 30 degrees from one another, each having a 2 mm axial depth and 6 mm transverse length. Each image had 10 *μ*m axial and 20 *μ*m transverse resolutions in tissue and consisted of 1024 axial pixels by 512 transverse pixels with a maximum scan velocity of 400 axial per second.

Cirrus HD-OCT images were generated using the Macular Cube 512 × 128 scan. Each image had 5 *μ*m axial and 10 *μ*m transverse resolutions in tissue and consisted of 512 × 128 volume cube. The scanning area measured 6 × 6 mm. The cube is composed of 128 horizontal examination lines of 512 A-scans each. The scanning speed is 27000 A-scans per second.

Topcon 3D-OCT 1000 images were generated using a 3D-scan, corresponding as for the Cirrus HD-OCT to the record of a cube of 6 mm per 6 mm composed of 128 horizontals examination lines of 512 A-scans each. Each image had 6 *μ*m axial and 20 *μ*m transverse resolutions in the tissue. Scanning speed is 18000 A-scans per second.

The main characteristics and acquisition protocol of each OCT instruments are listed in Table 1.

**Figure 1:**
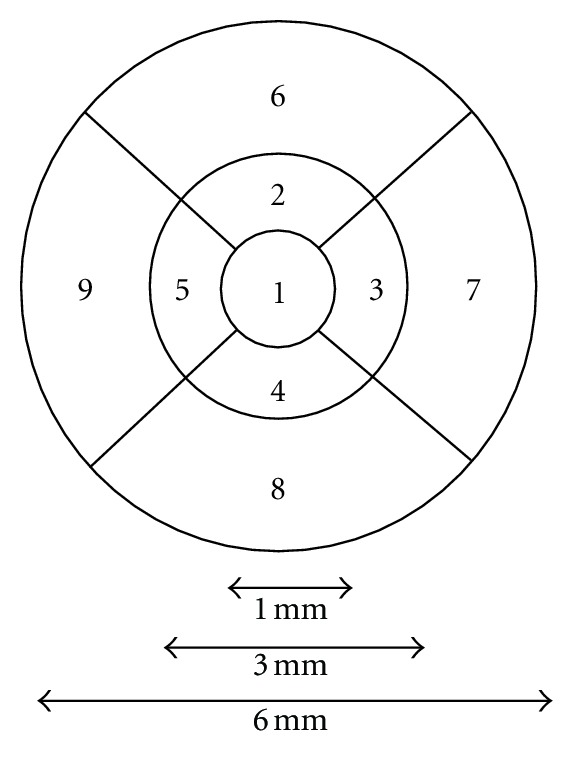
1 mm central retinal thickness area as described in the Early Treatment Diabetics Retinopathy Study (ETDRS).

The 1 mm central retinal thickness area as described in the Early Treatment Diabetics Retinopathy Study (ETDRS) fields corresponding to the CMT was compared in our study (Figure 1).

### 2.3. Statistical Analysis

Initial characteristics of subjects such as age and sex were described. Quantitative variables were expressed as mean ± standard deviation. The mean quality of the signal of each machine has been compared through an ANOVA table. Interoperator variability (between the 2 operators for each device) was studied using the Pearson correlation coefficient and the paired Student's *t*-test. Central macular thicknesses obtained from all OCT devices were compared one to each other using the paired Student's *t*-test. This comparison was also spotlighted by the Bland Altman plots construction [9].

Items were considered statistically significant if the probability value was *P* < 0.05. Statistical analyses were computerized using the STATA software, version 10.

## 3. Results

Thirty normal subjects aged from 19 to 57 years (34.4 ± 13.4) were included in the study (21 women, 9 men) or a sex ratio of 0.42. Measurement of CMT was possible in all 30 eyes.

All the measure records were automatic without operator manual adjustments as there was no segmentation error.

From instrument to instrument, the signal strength was significantly different (*P* < 0.05). The Stratus OCT, the Cirrus HD-OCT, and the Topcon 3D-OCT 1000 had a respective average signal strength of 5.3 ± 0.5, 8.6 ± 0.3, and 6 ± 0.4.

The differences in CMT ranged between 17.3 and 57.0 *μ*m. Compared with the time-domain Stratus OCT, Cirrus and Topcon SD-OCTs showed significantly higher CMTs. Difference in CMT measurements was 57.0 ± 8.1 *μ*m, 39.8 ± 9.5 *μ*m, and 17.3 ± 5.0 *μ*m between Stratus OCT and Cirrus HD-OCT, Stratus OCT and Topcon 3D-OCT 1000, and Cirrus HD-OCT and Topcon 3D-OCT 1000, respectively. The time-domain Stratus OCT had an average CMT of 197.5 ± 21.3 *μ*m, which is the thinnest compared to the Cirrus HD-OCT and Topcon 3D-OCT 1000 new generation OCTs. They had, respectively, an average CMT of 254.5 ± 23.3 *μ*m and 237.2 ± 23.5 *μ*m. Average CMT measured by each OCT was significantly different from one instrument to the other (*P* < 0.001) (Table 2).

**Figure 2:**
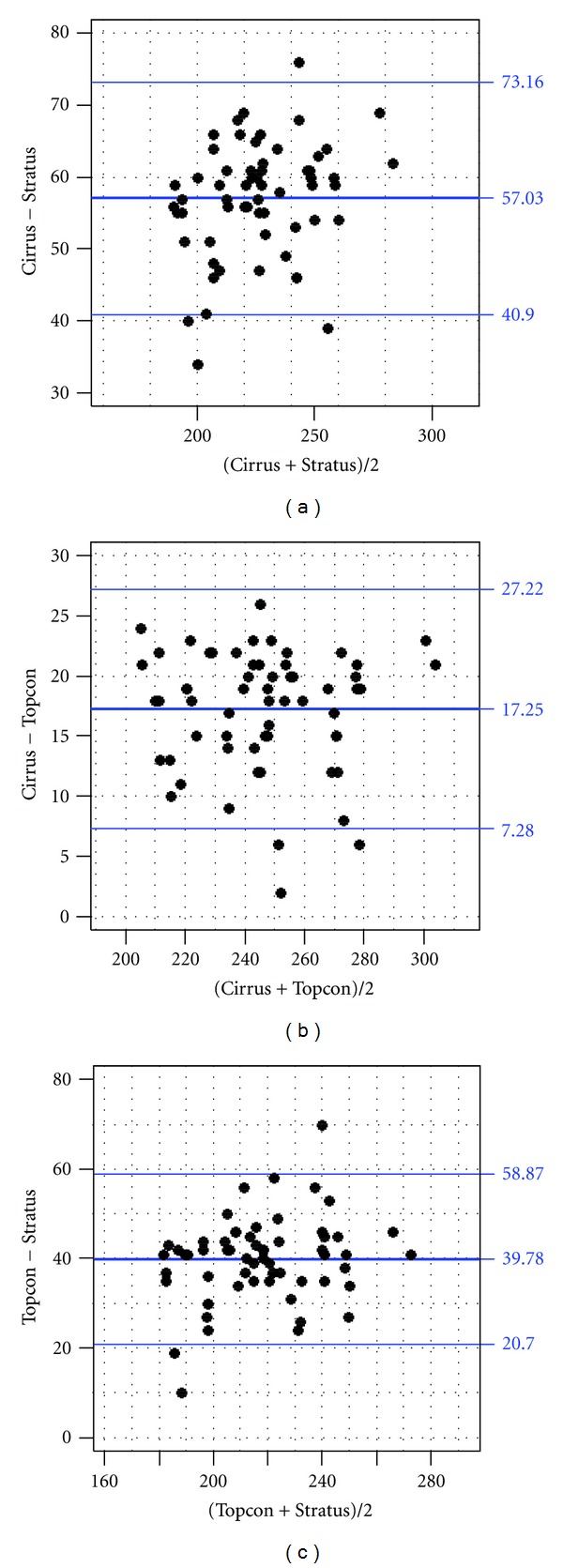
Bland Altman plots showing paired CMT differences versus average thickness for each machine comparison. (a) Cirrus minus Stratus. (b) Cirrus minus Topcon. (c) Topcon minus Stratus.

In addition for each instrument comparison, Bland-Altman plots displaying paired foveal thickness difference versus the average foveal thickness measurement of the two instruments are shown in Figures 2(a), 2(b), and 2(c).

The 95% limits of agreement for each comparison were 41.2 to 72.8 *μ*m for Cirrus-Stratus, 7.5 to 27.0 *μ*m for Cirrus-Topcon, and 21.1 to 58.5 *μ*m for Topcon-Stratus.

Table 3 shows CMT differences between the two operators obtained for each OCT device. We found a difference of 2 *μ*m for Stratus OCT and Cirrus HD-OCT and 3 *μ*m for Topcon 3D-OCT 1000. There was no statistical significant difference between measurements obtained by the operators since p values were, respectively, 0.164, 0.193, and 0.147 for Stratus OCT, Cirrus HD-OCT1, and Topcon 3D-OCT 1000. In our study the interoperator variability was very low. Moreover, a good correlation was found between the operator measurements with a correlation coefficient of 0.94 for the Stratus OCT, 0.98 for the Cirrus HD-OCT, and 0.96 for the Topcon 3D-OCT 1000.

## 4. Discussion

Until now, few studies had reported the interoperator variability of CMT measurement. In our study, we found no interoperator variability in CMT measurements using each OCT device on healthy patients. In 2004, Browning [10] studied interoperator variability in the CMT measurements obtained with Stratus OCT. Contrary to our study, his results showed a significant statistical difference between foveal zone measurements obtained by two operators. Measurements reliability depends certainly on eye movement during foveal focusing. Indeed, because of macular edema, Browning's patients have probably a worse foveal focusing during the exam than our healthy patients. However, Pierro et al. [11] found in a recent study a statistical interoperator variability on healthy subjects using various SD-OCT devices. Spectralis HRA+OCT and Cirrus HD-OCT presented the best operator-related results whereas 3D-OCT-1000 presented the worst interoperate-related reproducibility. According to the authors, fundus alignment and focusing who take longer with some devices can explain the results. Moreover the own peculiarities of each machine require particular operating competencies that may bring the examination either easier or more difficult to perform, thus influencing the precision of the result. It seems important that reducing interoperator variability as low as possible allows the best retina thickness monitoring, especially in age-related macular degeneration (AMD) disease, since retinal thickness is one of the retreatment's criteria. Indeed Framme et al. [12] evaluated interoperator variability with an SD-OCT device, in the indication of AMD retreatment after 3 injections of ranibizumab. The study showed that the interoperator variability seems to be of a limited concordance and then insufficient to decide on a retreatment.

In our study, we noted differing mean CMT from instrument to instrument. As in the main studies published, the lowest macular thickness was recorded with Stratus OCT. The highest value was recorded with Cirrus HD-OCT. All OCT software locates the inner retina boundary on the vitreoretinal interface or inner limiting membrane. The segmentation of the outer retinal boundary differs significantly from instrument to instrument [7, 13–15]. According to the manufacturers, segmentation depends on software algorithms different from one OCT device to the other. Many studies showed that in Stratus OCT, the inner segment/outer segment (IS/OS) interface of the photoreceptor layer is set as the posterior retinal boundary [16–18]. Already in 2005, Pierre-Khan et al. [13] compared OCT1 (Carl Zeiss, Meditec, Humphrey Division, Dublin Ca. USA) and Stratus OCT. Retinal thickness differed significantly from 25 *μ*m (+/− 26.2); the thickest measurement was given by Stratus OCT. A careful analysis of the segmentation showed that Stratus OCT uses the IS/OS line as the outer retinal boundary.

In our study, the highest CMT value was recorded with Cirrus HD-OCT. According to some authors the Cirrus HD-OCT outer retinal boundary is set as the OS photoreceptors and pigment epithelium junction. It means the Cirrus HD-OCT and Stratus OCT difference measured in our study (57 *μ*m) is the thickness of OS photoreceptors' layer. However other authors think that Cirrus HD-OCT may include pigment epithelium during the measurement [17]. Mylonas et al. [19] found two different outer retinal boundaries for the Cirrus HD-OCT and the Spectralis HRA+OCT. The last locates it on the level of the Bruch's membrane, and the Cirrus HD-OCT locates it within the pigment epithelium.

**Figure 3:**
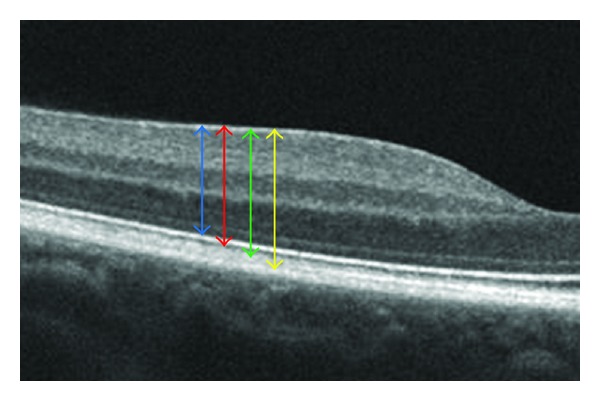
Example of high-definition cross-sectional image from a healthy volunteer allowing us to locate easily anterior and posterior retinal boundaries placement by Stratus, Topcon, Cirrus, and Spectralis. Blue arrow delimiting OCT retinal boundaries as inner limiting membrane (ILM) and IS/OS interface (Stratus), red arrow as ILM and OS/pigment epithelium junction (Topcon), green arrow as ILM and Verhoeff's membrane corresponding to the interdigitations of the external article layers of the photoreceptors in the pigment epithelium (Cirrus) and yellow arrow as ILM and Bruch's membrane (Spectralis).

Studies tend to confirm that Topcon 3D-OCT 1000, as all SD-OCTs, considers the pigment epithelium as its posterior segmentation limit. But in our study we found a significant difference of 17 *μ*m between Cirrus HD-OCT and Topcon 3D-OCT 1000. Mylonas et al. [19] also found a significant difference of 65.4 (±91.4) *μ*m between these two OCTs. The outer retinal boundary is probably not the same for Cirrus HD-OCT and Topcon 3D-OCT 1000. The difference could correspond to the outer article photoreceptors interdigitations in the pigment epithelium. The advent of very high-definition optical coherence tomography allows us to very precisely define the different retinal layers including the Verhoeff's membrane [20]. Indeed the localization of the outer retinal boundary of Cirrus HD-OCT at Verhoeff's membrane and the Topcon 3D-OCT 1000 one at the junction of photoreceptor external articles and of the pigment epithelium could explain our results and the literature's data (Figure 3).

Engelbert et al. [21] studied CMT obtained by Stratus OCT and Topcon 3D-OCT 1000 OCTs when using, as the outer retinal boundary, three different structures for thickness measurement among the IS-OS junction, the internal aspect of the RPE and Bruch's membrane. A good correlation was found between Stratus OCT and Topcon 3D-OCT 1000 OCTs in thickness measurement when using identical boundaries with greater measurements when using the RPE or the Bruch's membrane rather than IS-OS junction. CMT measurements will vary depending on the outer retinal boundary position defined by each instrument. These positions are regulated by the software of each instrument and have been chosen arbitrarily by the manufacturers.

Segmentation seems to be a fundamental element for retinal thickness determination, and the measurements reliability depends on its precision. Some of the instruments allow manual correction of the segmentation. Since we found no segmentation errors, we always used the automatic one. Mylonas et al. [19] investigated segmentation among normal patients and patients suffering from age-related macular Degeneration. The percentage of segmentation errors verges 0% for normal patients whatever the OCT device (Cirrus HD-OCT, Spectralis HRA+OCT, Topcon 3D-OCT 1000 and Stratus OCT). But it significantly differs from instrument to instrument in the population suffering from AMD. It is actually for 6% for the Cirrus HD-OCT, 27% for the Spectralis HRA+OCT, 32% for the Topcon 3D-OCT 1000, and 38% for the Stratus OCT. A recent study led by Querques et al. [22] showed a statistical difference in segmentation errors between Stratus OCT, Cirrus HD-OCT, and Spectralis HRA+OCT in a population of thirty-three patients with neovascular AMD. Cirrus HD-OCT showed fewer segmentation errors compared with Spectralis HRA+OCT and Stratus OCT.

Nevertheless these studies would not be sufficient to define the superiority of an OCT on another concerning the percentage of error of segmentation. It would be interesting to enlarge this type of study for collecting more data on the subject. Studies comparing Topcon 3D-OCT 1000 to other new generation OCTs are still rare, and studies have to be led.

In conclusion, the main result of our study is that central macular thickness value differs for each instrument depending on segmentation software, meaning no interchangeability of OCT devices for the retinal thickness measurement and followup. This problem can be solved maybe by using a conversion factor or looking to the outer retinal boundary detection for comparison of mean retinal values.

## Figures and Tables

**Table 1:** Description of acquisition protocols for each OCT device.

Instrument	Acquisition protocol
Stratus	Fast macular thickness scan
	Six radial scans (6 lines;
	128 A-scans per line)
	Scan area: 6 mm diameter circle
	Axial resolution (*μ*m): 10
	Transversal resolution (*μ*m): 20

Cirrus	Macular cube
	512 × 128 scans pattern (128 lines;
	512 A-scans per line)
	Scan area: 6 × 6 mm
	Axial resolution (*μ*m): 5
	Transversal resolution (*μ*m): 10

Topcon	3D scan
	512 × 128 scans pattern (128 lines;
	512 A-scans per line)
	Scan area: 6 × 6 mm
	Axial resolution (*μ*m): 6
	Transversal resolution (*μ*m): 20

**Table 2:** Comparison of central macular thickness averages obtained by each OCT instrument (ANOVA).

	Average O1/O2 (*μ*m) (IC 95%)
Stratus	198 (190.3–205.9)
Cirrus	254 (245.8–263.3)
Difference	**56 (52.3–60.7)**
*P*	**<0.001**

Stratus	198 (190.3–205.9)
Topcon	237 (228.5–246)
Difference	**39 (34.4–44.1)**
*P*	**<0.001**

Cirrus	254 (245.8–263.3)
Topcon	237 (228.5–246)
Difference	**17 (15.4–19.2)**
*P*	**<0.001**

**Table 3:** Comparison of central macular thickness obtained by each operator and each OCT instrument.

	Stratus (*μ*m)	Cirrus (*μ*m)	Topcon (*μ*m)
Operator 1 (O1)	196 (±20)	254 (±24)	237 (±24)
Operator 2 (O2)	198 (±23)	252 (±24)	234 (±23)
O1-O2	−2	2	3
Difference *P* > 0.05	0.164	0.193	0.147
